# ERCP in critically ill patients is safe and does not increase mortality

**DOI:** 10.1097/MD.0000000000028606

**Published:** 2022-02-04

**Authors:** Matthias Buechter, Antonios Katsounas, Fuat Saner, Guido Gerken, Ali Canbay, Alexander Dechêne

**Affiliations:** a Department of Gastroenterology and Hepatology, University Hospital Essen, University of Duisburg-Essen, Germany; b St. Nikolaus-Stiftshospital, Andernach, Germany; c Department of Medicine, University Hospital Knappschaftskrankenhaus Bochum, Ruhr University Bochum, Germany; d Department of General, Visceral, and Transplantation Surgery, University Hospital Essen, University of Duisburg-Essen, Germany; e Department of Gastroenterology, Hepatology and Endocrinology, General Hospital Nuremberg, Germany.

**Keywords:** endoscopic retrograde cholangiopancreatography, intensive care unit, simplified acute physiology score

## Abstract

Endoscopic retrograde cholangiopancreatography (ERCP) is the gold standard for minimally-invasive treatment of biliary or pancreatic tract disease. When treating patients on intensive care units (ICU) with ERCP, interventionalists are faced with considerably higher morbidity compared to patients in ambulatory settings. However, data on complications and outcome of critical ill patients undergoing emergency ERCP are limited.

A retrospective analysis of 102 patients treated on ICUs undergoing 121 ERCP procedures at the University Hospital of Essen, Germany between 2002 and 2016 was performed. Indications, interventional success, outcome including survival and procedure-related complications were analyzed. Patients’ condition pre-ERCP was categorized by using the “Simplified Acute Physiology Score” (SAPS 3).

66/102 patients (64.7%) were referred to ERCP from surgical ICU, 36/102 (35.3%) from nonsurgical ICU. The majority of patients were male (63.7%), the mean age was 54.1 ± 14.9 [21–88] years. Indications for ERCP were biliary complications after liver transplantation (n = 34, 33.3%), biliary leakage after hepatobiliary surgery (n = 32, 31.4%), and cholangitis/biliary sepsis (n = 36; 35.3%), respectively. 117/121 (96.7%) ERCPs were successful, 1 patient (1.0%) died during ERCP. Post-ERCP pancreatitis occurred in 11.8% of interventions. The median simplified acute physiology score 3 was 65 points, predicting a risk-adjusted estimated mortality of 48.8%, corresponding to an observed mortality of 52.2% (*P* = n.s.).

ERCP is safe in critically ill patients on ICU, it does not increase overall mortality rate and has a relatively low rate of procedure-associated complications.

## 1 Introduction

Since its first description by McCune et al^[1]^ in 1968, endoscopic retrograde cholangiopancreatography (ERCP) has become a safe and direct technique for evaluating pancreaticobiliary disease. In contrast to other imaging techniques, such as abdominal ultrasound (US), computed tomography (CT), magnetic resonance cholangiopancreatography (MRCP), and endoscopic ultrasound (EUS), which provide diagnostic information alone, ERCP offers therapeutic opportunities including gallstone removal, biliary decompression, and treatment of inflammatory strictures, leaks, and malignancies.^[2]^

Intensive care unit (ICU) patients present a difficult challenge in the diagnosis and treatment of diseases of the biliary or pancreatic tract. Altered mental status interferes with the patient's ability to communicate symptoms and the yield of physical examinations. Because of the high incidence of cholestasis in patients on ICU, laboratory parameters are often not specific and radiographic imaging studies lack sensitivity and specificity in evaluating biliary tract disorders.^[3]^ Given the high prevalence of multi-organ dysfunction, these patients present with high morbidity and mortality for ERCP when compared to the ambulatory setting.^[4]^ Having precise data on complications and outcome of patients on ICU undergoing emergency ERCP is important to decide for -or against an intervention in critically ill patients.

In this study, we aimed to check whether patients on ICU requiring ERCP have a higher mortality as compared to ICU patients who have no indication for ERCP, whether the specific indication for ERCP correlates to mortality, and the incidence of ERCP-related complications in this patient collective.

## 2 Patients and methods

### 2.1 Patients

This retrospective cohort study includes consecutive patients treated on ICU undergoing ERCP between 2002 and 2016. Out of a total of 102 patients, 66 (64.7%) were referred for ERCP from surgical ICUs, the remaining 36 (35.3%) from conservative ICU.

### 2.2 Patients’ condition before ERCP

Patients’ condition pre-ERCP was categorized by using the “Simplified Acute Physiology Score” (SAPS 3 Research Group, Wien, office@saps3.org). SAPS 3 is a widely-used scoring system for predicting risk-adjusted mortality in ICU patients after admission (Table 1).^[5,6]^ The SAPS 3 score was calculated for 69 out of 102 patients based on the parameters collected within the first 24 hours after admission on the ICU. A SAPS 3 calculator provided online as a software package for public download (http://www.saps3.org/resources-downloads/user-agreement/downloads/) was used to individually calculate the corresponding score for each patient. Analysis data, which included age, gender, Glascow Coma Scale scores, vital signs, and urine output were abstracted by trained ICU staff according to standard protocols. Based on mortality, patients were stratified into survivors and nonsurvivors.

**Table 1:** Variables used for calculation of SAPS 3.

Age
Length of stay before ICU admission
Intra-hospital location before ICU admission
Co-morbidities
Use of major therapeutic options before ICU admission: vasoactive drugs
ICU admission: planned or unplanned
Reason(s) for ICU admission
Surgical status at ICU admission
Anatomical site of surgery
Acute infection at ICU admission
Estimated GCS (lowest)
Total bilirubin (highest)
Body temperature (highest)
Creatinine (highest)
Heart rate (highest)
Leukocytes (lowest)
Hydrogen ion concentration (lowest)
Platelets (lowest)
Systolic blood pressure (lowest)
Oxygenation

GCS = Glasgow Coma Scale, ICU = intensive care unit, SAPS = simplified acute physiology score.

### 2.3 ERCP procedures

ERCP were conducted in the central endoscopy unit by senior endoscopists with a yearly caseload of at least 200 ERCP procedures. All patients were transported from ICU to the central endoscopy unit under supervision of an intensive care physician who maintained deep sedation in the patient during the intervention. ERCP was usually performed in the prone position with adducted arms to insure best fluoroscopic visualization and position to cannulate the papilla. If this position was not possible due to recent abdominal surgery (eg, drainages, wounds) the procedure was conducted with the patient in left lateral position or supine position. Fluoroscopy was performed with a high-end floor mounted flat panel detector (Artis zee, Siemens Healthineers, Germany).

### 2.4 Complications and mortality

Post-ERCP pancreatitis (PEP) normally is diagnosed when patients develop symptoms of acute pancreatitis (ie, abdominal pain) in addition to elevation of pancreatic enzymes in serum.^[7]^ In ICU patients, altered mental status and/or deep sedation interferes with the patient's ability to communicate symptoms and limits the sensitivity of physical examination. For study purposes, PEP was defined as lipase >3 times normal limit within 24 hours after ERCP (according to the revised European Society of Gastrointestinal Endoscopy (ESGE) guidelines published in 2014).^[8]^

Mortality was defined as death during hospital stay (in-hospital mortality, either on ICU or normal station). We also checked for patients who died during or less than 5 days after ERCP.

### 2.5 Statistical analysis

Statistical analyses were performed using GraphPad Prism, version 6.00 for Mac Os X (GraphPad Software, San Diego, CA). Categorial data was analyzed using the Chi-square test (calculation for multiple comparisons) and the Fisher exact test (calculation for single comparisons). Due to their abnormal distribution, descriptive data were shown as medians or percentages. Categorial factor analysis was performed using nonparametric tests to compare 2 groups. Overall, a *P*-value <.05 was considered statistically significant.

## 3 Results

### 3.1 Patient characteristics

Between 2001 and 2016, 121 ERCP procedures were performed in 102 patients treated on ICU (15 patients undergoing ≥ 2 ERCP). 76/102 patients (74.5%) were referred to ERCP from surgical ICU, 26/102 (25.5%) from nonsurgical ICU. The mean age was 54.1 ± 14.9 [21–88] years, patients were predominantly males (n = 65, 63.7%). Patients were mechanically ventilated in 95/121 ERCP procedures (78.6%) and received vasopressors (eg, norepinephrine and/or vasopressin) in 87/121 interventions (71.9%). Sedation was performed by use of propofol, midazolam or dexmedetomidine. SAPS 3 were calculable for 69/102 patients. The median SAPS 3 was 65, corresponding with an estimated mortality of 48.8%. The mean hospitalization on ICU was 29.3 ± 30.5 [1–150] days. Patient characteristics are listed in Table 2. Indications for ERCP were suspicion of biliary complications after liver transplantation (LT) (n = 34, 33.3%), biliary leakage after hepatobiliary surgery (n = 32, 31.4%), and cholangitis/biliary sepsis (n = 36; 35.3%). ERCP indications and results are demonstrated in Table 3.

**Table 2:** Patient characteristics (n = 102).

Age (yr)	54.1 ± 14.9 [21–88]
Gender	
Male	65 (63.7%)
Female	37 (36.3%)
SAPS 3	65 [35–110]
ICU stay (d)	29.3 ± 30.5 [1–150]
Intervention time (min)	50.2 ± 31.3 [10–181]
Patients undergoing ≥ 2 ERCP	15 (14.7%)
Technical success	117/121 (96.7%)
Referring unit	
Surgical ICU	66 (64.7%)
Nonsurgical ICU	36 (35.3%)

ERCP = endoscopic retrograde cholangiopancreaticography; ICU = intensive care unit; SAPS 3 = simplified acute physiology score.

**Table 3:** ERCP results according to indication (n = 102).

Indication	Endoscopic findings	
Biliary complications after LT (n = 34)	Biliary leakage	15 (44.1%)
	Anastomotic stricutures	13 (38.2%)
	Nonanastomotic strictures	3 (8.8%)
	Normal biliary tract	2 (5.9%)
	No endoscopic access	1 (2.9%)
Biliary leakage liver resection (n = 32)	Biliary leakage	26 (81.3%)
	Normal biliary tract	5 (15.6%)
	No endoscopic access	1 (3.1%)
Cholangitis/cholangiosepsis (n = 36)	Cholelithiasis and/or cholangitis	23 (63.9%)
	Sclerosing cholangitis	7 (19.4%)
	Normal biliary tract	5 (13.9%)
	No endoscopic access	1 (2.8%)

RCP = endoscopic retrograde cholangiopancreatography, LT = liver transplantation.

### 3.2 Diagnostic results and therapeutic interventions

The mean duration of the intervention was 50.2 ± 31.3 [10–181] minutes. ERCP was successful in 117/121 (96.7%) cases. Overall endoscopic findings were 45 biliary leakages (37.2%), 25 choledocholithiasis/cholangitis (20.7%), 16 normal cholangiograms (13.2%), 16 biliary complications after LT (13.2%; 13 anastomotic strictures, 3 ischaemic-type biliary lesions, 12 secondary sclerosing cholangitis [9.9%]), 4 unsuccessful interventions (3.3%; major duodenal papilla not detectable, bile duct not cannulated), and 3 papillary or bile duct bleedings (2.5%), respectively. Therapeutic interventions performed during ERCP were in detail: endoscopic sphincterotomy (EST) (n = 74), implantation/removal of nonexpanding plastic stent (n = 69), balloon dilatation (n = 33), implantation/removal of self-expanding metal stent (n = 3).

### 3.3 Indication, ERCP related complications, and mortality according to SAPS 3

One out of 102 patients deceased during the procedure (mortality < 1.0%). ERCP-related re-interventions were required in 3/121 (2.5%) cases. These complications were bleeding after EST in 2/74 patients receiving EST (2.7%) and stent dislocation in 1/69 patients receiving nonexpanding plastic stent (1.4%). PEP occurred in 12/102 patients (11.8%) without PEP-associated mortality. In case of increased risk for PEP (eg, multiple cannulation or contrast medium application to the pancreatic duct, EST) 100 mg of rectal indomethacin was immediately administered after ERCP in all patients without contraindications to nonsteroidal antiinflammatory drug administration.

**Figure 1:**
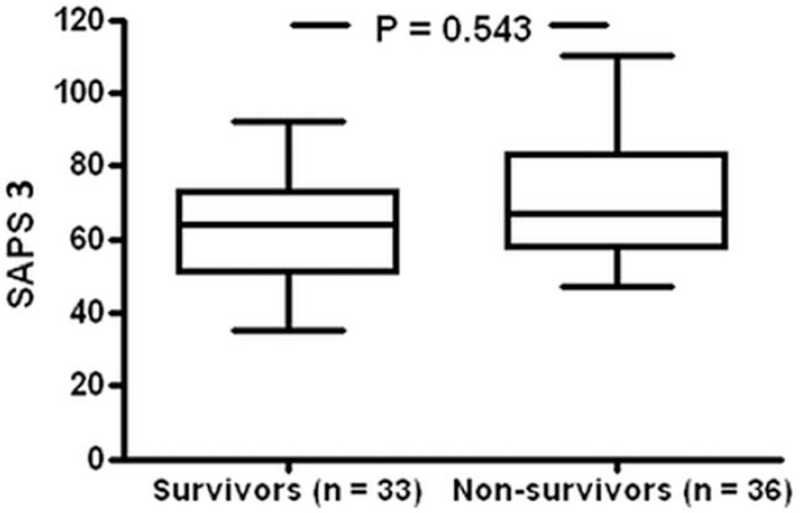
SAPS 3 values of 69 patients were subjected to statistical analysis in order to screen for significant baseline differences in estimation of severity of disease between survivors and nonsurvivors. A two-tailed *t* test detected no statistical differences in SAPS 3 between both patient groups.

**Figure 2:**
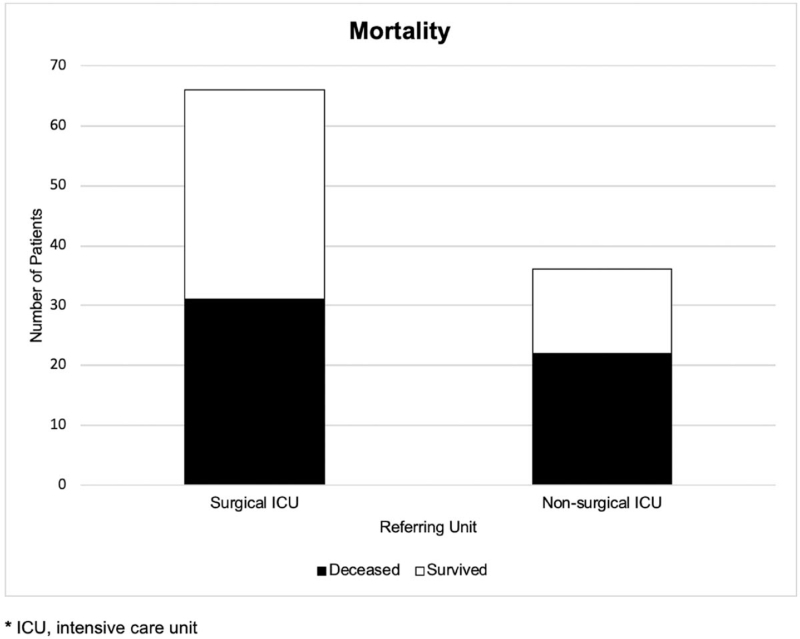
Mortality of ICU patients with ERCP (n = 102) stratified by referring unit (*P* = n.s.).

SAPS 3 was calculable in 69/102 patients with a median score of 65 [35–110] points. Predicted mortality calculated with the SAPS 3 scoring system of 48.8% or 33.6 patients was very close to the observed mortality rate of 52.2% or 36 patients. In consistence with this result, there was no statistical difference between the SAPS 3 scores (Fig. 1). Mortality among all patients (n = 102) was 52.0%, meaning 53 patients deceased intra-ERCP (1/53) or on ICU/normal station (52/53). For all deceased patients, the mean timespan between ERCP and death was 28.3 ± 35.1 [0–173] days. 8/102 (7.8%) patients died within 5 days after ERCP. Comparing mortality of patients from surgical ICU to patients from nonsurgical ICU undergoing ERCP displayed a nonsignificant trend to higher mortality rate in nonsurgical ICU patients (mortality surgical ICU 47.0% [31/66 patients], nonsurgical ICU 61.1% [22/36 patients], *P* = n.s.) (Fig. 2). In surviving patients, the mean time between ERCP and discharge from hospital was 35.7 ± 30.1 [4–140] days.

**Figure 3:**
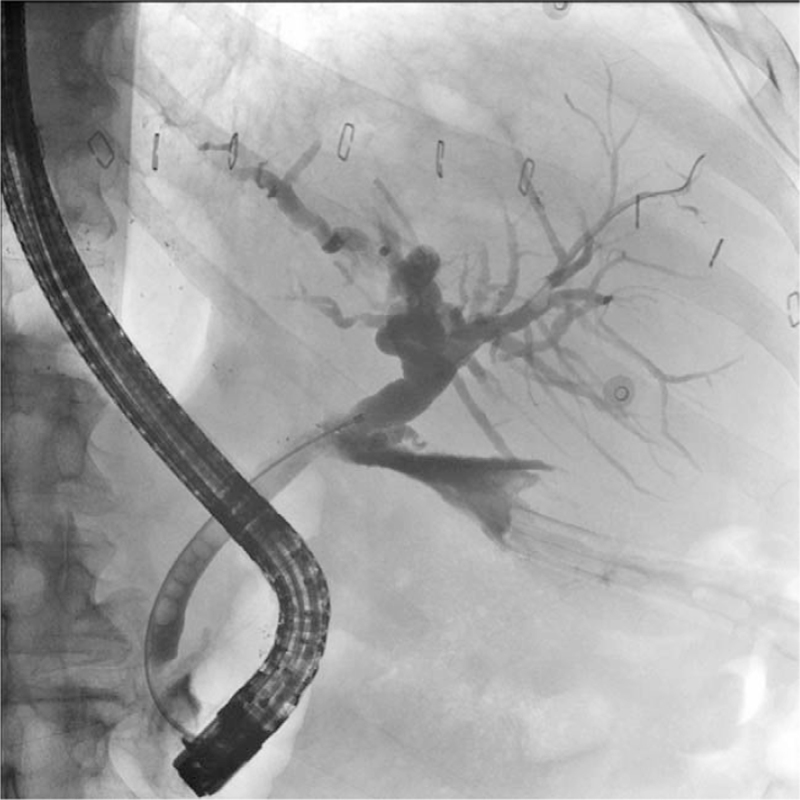
Cholangiogram of a male patient with bile leak at the biliary anastomosis after liver transplant.

In 34/102 (33.3%) patients, ERCP was performed for suspected biliary complications after LT. ERCP showed biliary leakage in 15 (44.1%) (Fig. 3), anastomotic strictures in 13 (38.2%), nonanastomotic strictures in 3 (8.8%), and a regular biliary tract in 2 of these patients (5.9%). Endoscopic access was not obtained in 1 patient (2.9%). The mean time between LT and ERCP was 31.2 ± 24.5 [2–102] days. The mortality in this subgroup was 18/34 (52.9%).

In 32/102 (31.4%) patients, ERCP was performed for suspected bile leaks after hepatobiliary surgery (multisegmental resections in all patients). ERCP confirmed bile leaks in 26 (81.3%) and showed a regular postoperative biliary tract in 5 (15.6%) patients. Endoscopic access was not obtained in 1 patient (3.1%). The mean time between surgery and ERCP was 11.6 ± 13.8 [0–66] days. Mortality was 13/32 (40.6%) in this cohort.

**Figure 4:**
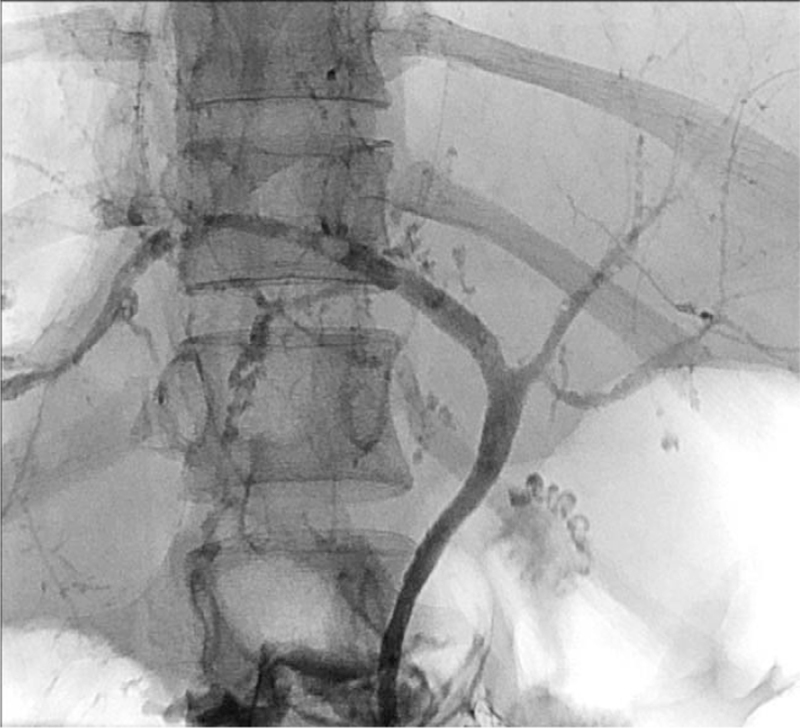
Cholangiogram of a female patient suffering from “sclerosing cholangitis in critically ill patients” (SC-CIP) following polytrauma and long-term intensive care treatment including mechanical ventilation.

In 36/102 (35.3%) patients, ERCP was performed following clinical signs suggesting cholangitis. ERCP revealed cholelithiasis and/or cholangitis in 23 (63.9%) and a regular biliary tract in 5 (13.9%) patients. Sclerosing cholangitis was found in 7/36 patients (19.4%) (Fig. 4). Endoscopic access was not obtained in 1 patient (2.8%). The mortality in this subgroup was 22/36 (61.1%).

**Figure 5:**
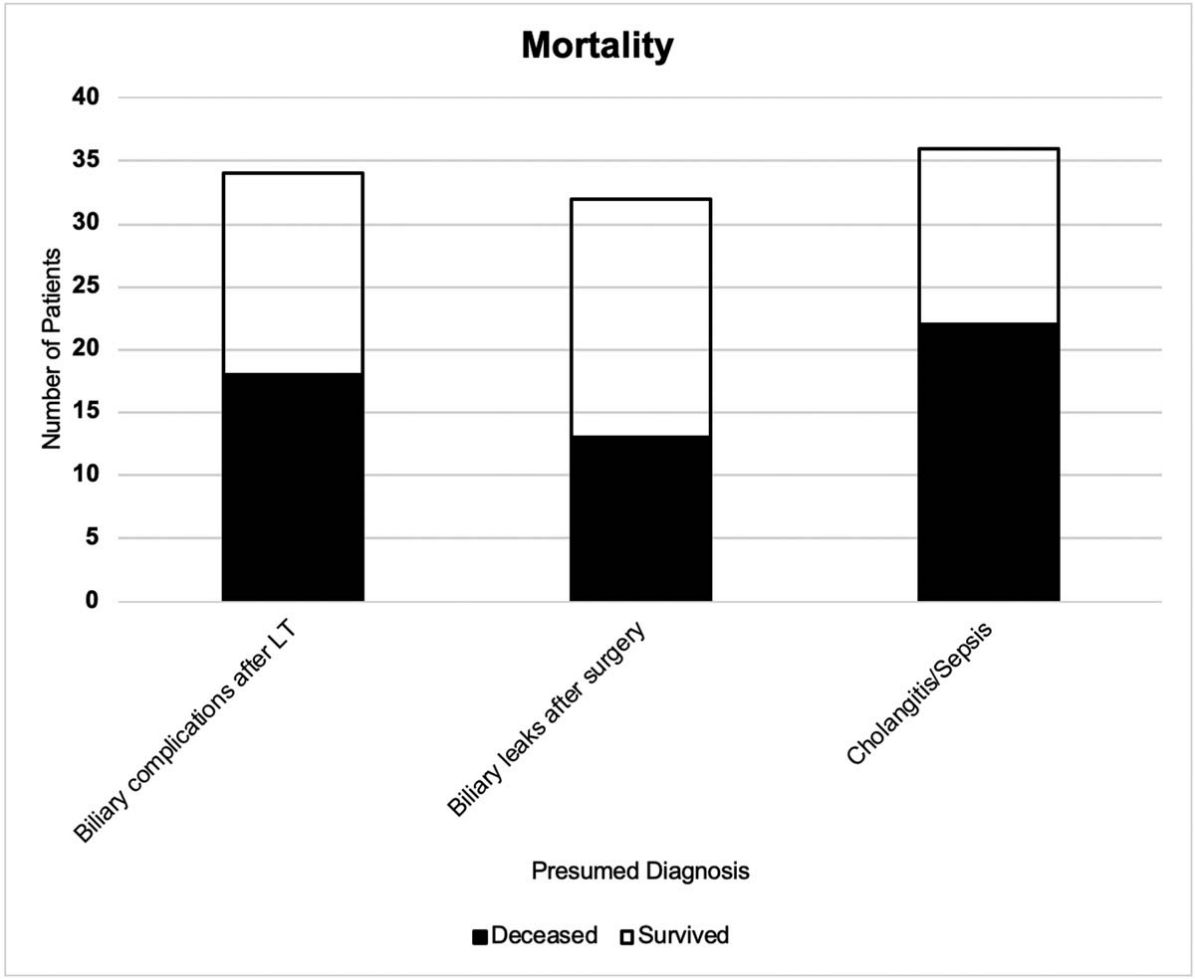
Mortality of ICU patients with ERCP (n = 102) stratified by indication (*P* = n.s.).

Statistical analysis showed no significant differences in mortality between groups of patients with regard to indication of ERCP (*P* = n.s.) (Fig. 5).

## 4 Discussion

ERCP is accepted as a safe technique for evaluating and treating pancreaticobiliary disease. Since the first EST in 1974, its role has evolved from a diagnostic procedure to one that is almost exclusively therapeutic. In contrast to other imaging techniques (ie, US, CT, MRCP, and EUS) that provide important diagnostic information and allow appropriate selection of patients, ERCP is the main minimally-invasive procedure offering therapeutic options with a number of well-evaluated interventional techniques.^[2,9,10]^

In a nonintensive care setting, ERCP is a safe examination with a relatively low, yet well-defined complication rate.^[11]^ In contrast, data on safety and efficiency of emergency ERCP performed in critically ill patients treated on ICU are still limited. This patient collective presents a difficult challenge to any endoscopic team, not least because of high expected mortality and the risk of further deterioration in case ERCP-related complications occur.

Overall mortality after diagnostic ERCP in non-ICU patients is approximately 0.2% and twice as high after therapeutic ERCP (0.4%–0.5%).^[12–15]^ However, the mortality rate must be regarded in the context of patient comorbidity and the indication for ERCP. Among 102 patients undergoing 121 ERCP procedures in our cohort, 1 patient died from septic shock during ERCP (intrainterventional mortality < 1.0%). Overall mortality of ICU patients undergoing ERCP, defined as in-hospital mortality, was 52.0%. Calculating the estimated mortality by using SAPS 3 (48.8%), statistical analysis revealed no significant difference between estimated and observed mortality, respectively. In the deceased patients, the mean timespan between ERCP and death was 28.3 ± 35.1 [0–173] days. A direct chronological relation between ERCP procedures and mortality, defined as death ≤5 days after ERCP, was found in 8/102 patients (7.8%). None of these patients suffered from ERCP-related complications. However, statistically significant benefit of ERCP procedure regarding mortality could not be demonstrated, which might be explained by the relatively small sample size and the missing control group.

The most common serious ERCP complication is pancreatitis. Although methods of reducing PEP, such as patient selection, pharmacological prophylaxis and modifications in technique have been described, it is reported to occur in 2% to 10% of unselected patients, ranging from 2% to 4% in low-risk situations up to 8% to 40% in high-risk patients.^[7,12,16–21]^ According to ESGE guidelines, the diagnosis of PEP requires 2 of the 3 following criteria: abdominal pain consistent with acute pancreatitis (acute onset of a persistent, severe, epigastric pain often radiating to the back); serum lipase or amylase activity at least 3 times greater than the ULN; and characteristic findings of acute pancreatitis on contrast-enhanced CT and, less commonly, magnetic resonance imaging or abdominal ultrasonography.^[8]^ ICU patients present a difficult challenge when applying this definition, particularly in regard to abdominal pain consistent with acute pancreatitis and serum lipase or amylase activity at least 3 times greater than the ULN, because altered mental status (eg, sedation during mechanical ventilation) interferes with the patient's ability to communicate symptoms. Therefore, we defined PEP according to ESGE guidelines as elevation of serum lipase at least 3 times upper ULN 24 hours after ERCP. 11.8% of ICU patients with ERCP were positive for this definition, matching with the high-risk population published in the literature. Nevertheless, this analysis has some limitations, since a transient increase in serum pancreatic enzymes occurs in up to 75% of ERCP-patients, not necessarily implying pancreatitis.^[12,16]^ Furthermore, abdominal CT scan and additional determination of serum amylase, which could possibly improve the diagnostic accuracy, were not routinely performed in our collective. In how far PEP led to further clinical deterioration or prolongued ICU stay is only speculative and could not be adequately evaluated by our retrospectively analyzed data. A certain relationship between PEP and mortality could not be revealed.

Other ERCP-related complications, such as hemorrhage after EST (1.3%), perforation (0.1%-0.6%), new onset of biliary infection (<1%) or cardiopulmonary complications (1%) are less frequent.^[12,13,15,22–24]^ Compared to published data, the incidence of ERCP-related re-interventions among ICU patients was slightly higher in our collective (2.5%). Hemodynamically relevant bleeding after EST was seen in 2/74 cases (2.7%), while stent dislocation requiring re-ERCP occurred in 1/69 (1.4%). None of our patients suffered from ERCP-induced perforations.

The indications for ERCP in ICU patients are distinctly different than in non-ICU patients. The most common indications for the ERCP in the ambulatory setting are mechanical jaundice caused by choledocholithiasis, malignant lesions of the pancreatic-duodenal region (bile ducts, duodenal papilla, and pancreatic head), and inflammatory strictures of the bile duct.^[25]^ Our data shows that ICU patients provide a different spectrum of biliary disease with the majority of these patients being referred to ERCP from operative ICU for complications after LT or resection. This patient collective also poses a challenge for the interventionalist since the standard position (prone position with adducted arms) is not always possible due to recent abdominal surgery. Furthermore, anatomic conditions and oedema of the duodenal region play a role after previous surgery. In case of ERCP failure (4 patients (3.3%) had failed ERCP in our study), sonographically guided percutaneous transhepatic biliary drainage can performed as second-line approach if dilated bile ducts are present, which was the treatment of choice in our patient collective. As an alternative, EUS guided drainage of the biliary tract is gaining attention over the last years. However, this procedure is not routinely performed in our center.

In 34/102 (33.3%) patients, ERCP was performed for suspected biliary complications after LT. Approximately 30% of patients after LT suffer from biliary tract complications resulting in significant increase of morbidity and mortality.^[26,27]^ The most common biliary complications following LT are bile leaks (2%–21%) and strictures (anastomotic [6%–34%] or nonanastomotic [0.5%–10%] strictures, also called ischemic type biliary strictures).^[28–32]^ Endoscopic management currently is the primary approach for biliary complications after LT.^[27,33,34]^ In our cohort, biliary leaks were found in 44.1% of ICU patients undergoing ERCP for complications after LT, followed by anastomotic (38.2%) and nonanastomotic strictures (8.8%). In 32/102 patients (31.4%), ERCP was indicated to investigate bile leaks after hepatobiliary surgery (mainly liver resections). Despite improvements in surgical techniques and postoperative patient care, bile leaks remain a major problem after liver resection with reported occurrence rates from 3% to 12%.^[35,36]^ The International Study Group of Liver Surgery defines relevant bile leak as a bilirubin concentration in the drainage fluid at least 3 times the serum bilirubin concentration on or after the third postoperative day.^[35]^ Endoscopic therapy is sufficient in approximately 90% of these patients and should be considered first-line therapy.^[34,36,37]^ Likewise, ERCP was effective in detection and treatment of bile leaks after hepatobiliary surgery in 96.9% of our patients.

Acute cholangitis is a bacterial infection of the biliary tract predominantly caused by biliary obstruction due to choledocholithiasis. This medical emergency is potentially lethal when progressing to cholangiosepsis, and prompt risk stratification is mandatory to facilitate timely treatments and improve clinical outcomes.^[38]^ Despite improvements in therapy, mortality of biliary sepsis is still high, reaching 40%.^[39–41]^ In 36/102 patients (35.3%) in our collective, ERCP was indicated for suspicion of acute cholangitis and/or biliary sepsis, with a high mortality of 61.1% in this collective. Sclerosing cholangitis (synonymous with sclerosing cholangitis in critical ill patients) was found in 7/36 (19.4%) patients. This disease occurs in association with intensive-care treatment of patients with major surgery, trauma, burns, and other life-threatening events. It is a progressive cholestatic liver disease characterized by inflammation-mediated necrosis of the biliary epithelium and formation of biliary casts, leading to rapid destruction of predominantly intrahepatic bile ducts.^[42,43]^ By means of ERCP, biliary cast formations can be extracted and inflammatory strictures can be treated with balloon dilatation or bougienage to slow progression of liver failure. Nevertheless, LT remains the only curative treatment option. Correspondingly, observed mortality among patients with sclerosing cholangitis was high in our collective (42.9%), as expected. Interestingly, there was no significant difference in mortality between groups of patients with regard to indication of ERCP (Fig. 3). Though there was a trend to higher mortality in patients referred to ERCP from nonsurgical ICU (61.1%) than from surgical ICU (47.0%) as expected, statistical analysis revealed no significant difference (*P* = n.s.) (Fig. 2).

Of course, we are aware of the limitations of our study, the most important of them being its retrospective character and the relatively small cohort size. Also, the study was performed as a single-center observational study and a valid control group was not available. However, larger multi-center studies are warranted for confirming of our results.

## 5 Conclusions

ERCP is safe even in critically ill patients treated on ICU, as reflected by SAPS 3. The technical success rate is very high with an incidence of procedure-related complications comparable to the non-ICU setting. Short- and long-term mortality is high in these patients as expected, but is not increased by ERCP. The difference in preinterventional morbidity and indications for ERCP should be taken into account when comparing to results of biliary interventions on non-ICU patients.

## Acknowledgments

We acknowledge support by the Open Access Publication Fund of the University of Duisburg-Essen.

## Author contributions

**Conceptualization:** Fuat Saner, Guido Gerken, Ali Canbay, Alexander Dechene.

**Data curation:** Matthias Buechter, Antonios Katsounas.

**Formal analysis:** Matthias Buechter, Antonios Katsounas.

**Investigation:** Fuat Saner, Alexander Dechene.

**Methodology:** Ali Canbay, Alexander Dechene.

**Project administration:** Matthias Buechter, Alexander Dechene.

**Software:** Matthias Buechter.

**Supervision:** Ali Canbay, Alexander Dechene.

**Validation:** Alexander Dechene.

**Writing – original draft:** Matthias Buechter.

**Writing – review & editing:** Guido Gerken, Ali Canbay, Alexander Dechene.
